# Anticancer Effect of Tanshinones on Female Breast Cancer and Gynecological Cancer

**DOI:** 10.3389/fphar.2021.824531

**Published:** 2022-01-25

**Authors:** Zhou Jin, Yu Chenghao, Peng Cheng

**Affiliations:** ^1^ State Key Laboratory of Southwestern Chinese Medicine Resources, Chengdu University of Traditional Chinese Medicine, Chengdu, China; ^2^ Basic Medical College, Chengdu University of Traditional Chinese Medicine, Chengdu, China; ^3^ College of Pharmacy, Chengdu University of Traditional Chinese Medicine, Chengdu, China

**Keywords:** gynecological cancer, tanshinones, molecular mechanism, traditional Chinese medicine, female breast cancer

## Abstract

Female breast cancer, ovarian cancer, cervical cancer, and endometrial cancer are the most common tumors and the most common causes of cancer-related mortality worldwide in women. Drugs derived from natural plants play important roles in malignant tumor therapy. *Salvia miltiorrhiza* is a commonly used Chinese herb which has been used in the treatment of liver diseases and cardiovascular diseases because of its positive effect of promoting blood circulation, increasing oxidative stress, and removing blood stasis. Recently, studies have found that fat-soluble components of *Salvia miltiorrhiza* such as tanshinone II, tanshinone I, cryptotanshinone, and dihydrotanshinone I displayed good antitumor activity *in vivo* and *in vitro* for gynecological cancer by different molecular mechanisms. In this study, the latest research progress on the antitumor effect and mechanism of tanshinone compounds in breast cancer and gynecological cancer was reviewed to provide references for the research and clinical application of these compounds (tanshinone II, tanshinone I, cryptotanshinone, and dihydrotanshinone I).

## Introduction

Female breast cancer, ovarian cancer, cervical cancer, and endometrial cancer are the most common tumors and the most common causes of cancer-related mortality worldwide in women. Surgical resection, radiotherapy, and chemotherapy remain effective curative treatments for early-stage gynecological tumors (DeSantis et al., 2014; Yadav et al., 2017; Amant et al., 2019; Coughlin, 2019). Unfortunately, only a minority of patients are candidates for surgical resection. Systemic chemotherapy has been widely employed in clinical treatment of female breast cancer and gynecological tumors, which increases the median survival time of patients with advanced gynecological tumors (Ponde et al., 2019; Burstein, 2020; Tsuyoshi et al., 2020). The commonly used chemotherapy drugs include natural anticancer agents from herb, alkylating agents, antimetabolites (methotrexate and cyclophosphamide), antitumor antibiotics, and platinum analogs (Gu et al., 2016; Kim et al., 2020; Li et al., 2020; Liu et al., 2020). Drugs derived from natural plants play important roles in malignant tumor chemotherapy. Therefore, screening new anticancer plant medicine has become one of the hotspots for tumor therapy in recent years.

Traditional Chinese medicine (TCM) possesses a long history and a lot of successful experiences. Lots of traditional Chinese medicines such as Radix Kansui, *Rheum rhabarbarum*, and Cinobufotalin can be used to inhibit breast cancer *in vitro* and *in vivo* by regulating differentially expressed genes and signaling pathways (Li J. et al., 2019; Liu YT. et al., 2019; Yang Z. et al., 2021). Many compounds or monomers from TCM such as oridonin, berberine, and matrine have displayed some antitumor effects *in vitro* and *in vivo* by inhibiting the proliferation of various cancer cells.

*Salvia miltiorrhiza* is a commonly used Chinese herb. It is believed in TCM that *Salvia miltiorrhiza* is good at promoting blood circulation and removing blood stasis. Li Shizhen said that *Salvia miltiorrhiza* has an effect in Siwu decoction in the Compendium of Materia Medica. *Salvia miltiorrhiza* is mainly produced in Sichuan, Shanxi, Hebei, Jiangsu, and Anhui, and its medicinal parts are roots and rhizomes. In the National Pharmacopoeia (2020 Edition), the efficacy of *Salvia miltiorrhiza* is summarized as removing blood stasis and alleviating pain, promoting blood circulation and dredging meridians, and clearing heart and eliminating annoyance. It is used for irregular menstruation, amenorrhea, accumulation of symptoms, chest and abdomen tingling, heat arthralgia pain, sore swelling, restlessness, hepatosplenomegaly, and angina pectoris. *Salvia miltiorrhiza* has been used in the treatment of liver diseases and cardiovascular diseases because of its positive effect of promoting blood circulation and removing blood stasis.

**FIGURE 1:**
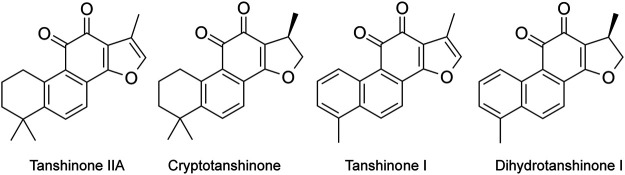
Main fat-soluble components in *Salvia miltiorrhiza* (Tanshinone IIA, cryptotanshinone, tanshinone I, and dihydrotanshinone I).

The effective components of *Salvia miltiorrhiza* include fat-soluble and water-soluble components. Fat-soluble components include tanshinone I, dihydrotanshinone I, tanshinone IIA, and tanshinone IIB (Figure 1). Water-soluble (phenolic acids) components include Danshensu, salvianolic acid A, salvianolic acid B, purple oxalic acid, protocatechuic aldehyde, and rosmarinic acid. Modern pharmacological studies have shown that these bioactive components have anti-inflammatory, anti-oxidative stress, antitumor and anti-myocardial ischemia effect, and can inhibit left ventricular hypertrophy, dilate blood vessels, resist atherosclerosis, protect brain tissue, resist thrombosis, improve microcirculation, promote fracture healing, significantly inhibit pulmonary fibrosis, and activate immunity.

In recent years, it has been found that fat-soluble components, namely, tanshinones, have good antitumor activity *in vivo* and *in vitro*. In this study, the latest research progress on the antitumor effect and mechanism of tanshinone compounds in breast cancer and gynecological cancer was reviewed to provide references for the research and clinical application of these compounds.

## Tanshinones Inhibit Cell Proliferation

In breast cancer, tanshinone IIA significantly inhibits colony formation and BrdU incorporation by a dose- and time-dependent inhibitory effect on MCF-7 cells (IC50 = 0.25 mg/ml), and tumor growth in negative human breast IDC-xenografted animal model, which is attributed to apoptosis induced through upregulation and downregulation of multiple genes involved in cell proliferation and apoptosis (Wang et al., 2005; Fu et al., 2014). Other studies have shown that tanshinone IIA significantly downregulated the viability of ER-positive human breast cancer cells (MCF-7) and ER-negative human breast cancer cells (MDA-MB231) by dose- and time-dependent manners *in vitro* through downregulating the expression of P53 and Bcl-2 (Lu et al., 2009). According to another study, tanshinone IIA inhibits the growth of three kinds of breast cancer cells, such as MDA-MB453, SKBR3, and MDA-MB231 cell lines (Gong et al., 2012). Su et al. found that tanshinone IIA suppresses tumor growth in MDA-MB-231 xenografted animal model by decreasing Erb-B2 and NF-κBp65 expression, and increasing caspase-3 expression and Bax/Bcl-xL ratio (Su and Lin, 2008; Su et al., 2012). Yan et al. showed that tanshinone IIA blocks cell proliferation and increased cell apoptosis on human breast cancer BT-20 cells by activating ER stress and MAPK pathway (increasing caspase-12, GADD153, and phospho-p38 protein expression and downregulating Bcl-xL and phospho-ERK expression) (Yan et al., 2012). Nicolin et al. also found that tanshinone IIA inhibits the proliferation of MCF-7 and MD-MB-231 cells by inducing cell apoptosis and blocking cell cycle (Nicolin et al., 2014). Tanshinone I is one of the vital fat-soluble monomer components from the traditional herbal medicine, *Salvia miltiorrhiza* Bunge (Wang et al., 2019; Zhou et al., 2020a). It has been found that Tanshinone I has a cytotoxicity effect on breast cancer cells in a dose-dependent manner. The molecular mechanisms demonstrated that tanshinone I induces G0/G1 phase arrest in MCF-7 cells and both S and G2/M phase arrests in MDA-MB-231 cells by downregulating cell cycle markers such as cyclin D, CDK4, and cyclin B protein levels, and promoted cell apoptosis by increasing the protein level of pro-apoptosis–related proteins [cleaved PARP (c-PARP)] and reducing anti-apoptosis–related protein (Bcl-2) (Gong et al., 2012). Tanshinone I inhibits the proliferation of MDA-MB-231 cells and MCF-7 cells, and induces cell apoptosis by decreasing the Bcl-2/Bax ratio and activating cleavage of caspase-3 in cancer cells (Nizamutdinova et al., 2008b). Tanshinone IA also weakens cell proliferation by inhibiting DNA synthesis, inducing apoptosis, and arresting cell cycle of ER-negative human breast cancer cells (Jing et al., 2007). In summary, tanshinones can inhibit the growth of breast cancer cells by inducing cell apoptosis and cell cycle arrest *in vitro* and *in vivo*. At the molecular level, tanshinones can promote pro-apoptotic protein expression, and reduce anti-apoptotic protein expression and cell cycle–related protein expression.

In ovarian cancer, tanshinone IIA significantly inhibits cell growth and aggressiveness by inducing cell apoptosis, which is mediated by the PI3K/AKT/JNK signaling pathway in ovarian cancer (Zhang et al., 2019). Other studies showed that tanshinone IIA has a growth inhibitory effect by inducing cell apoptosis and downregulating survivin expression in ovarian cancer cells (Jiao and Wen, 2011; Li N. et al., 2018). Our studies showed that tanshinone IIA inhibits the growth of ovarian cancer cells in a dose-dependent manner by inducing G2/M phase arrest and cell apoptosis (Zhou et al., 2020c). In ovarian cancer, tanshinone IA significantly suppresses cell proliferation *in vitro* and tumor growth *in vivo* by inducing apoptosis and promoting autophagy *via* the inactivation of the PI3K/AKT/mTOR pathway in ovarian cancer (Zhou et al., 2020a). Another bioactive component dihydrotanshinone I significantly inhibits the proliferation of ovarian cancer cells *in vitro* and *in vivo* by modulating the PI3K/AKT signaling pathway (Wang et al., 2020). Cryptotanshinone inhibits cellular glycolysis–induced cell growth and proliferation by suppressing the STAT3/SIRT3/HIF-1α signaling pathway (Yang et al., 2018).

In cervical cancer, tanshinone IIA inhibits cell viability, resulting in a 72.7% reduction in tumor volume *in vivo* by inducing apoptosis and decreasing glycolysis (Liu Z. et al., 2019). It is reported in other studies that tanshinone IIA inhibits the proliferation of human cervical cancer CaSki, SiHa, HeLa, and C33a cells *in vitro*, leading to a 66% reduction in the volume of cervical cancer xenograft *in vivo* by inducing S phase cell cycle arrest and p53-mediated apoptosis (Munagala et al., 2015). Tanshinone IIA inhibits the proliferation of HeLa cells by inducing pyroptosis *via* upregulating the expression of GSDMD and miR-145 (Tong et al., 2020). Two proteomic analyses revealed that tanshinone IIA possesses strong growth inhibition effect against human cervical cancer cells in dose- and time-dependent manners by regulating the expressions of proteins involved in microtubule assembly, leading to G (2)/M phase arrest, mitochondria-mediated intrinsic apoptosis, and endoplasmic reticulum stress (Pan et al., 2010; Pan et al., 2013). Tanshinone I suppresses the proliferation by inhibiting ELK1 and downregulating KRAS-AKT axis in cervical cancer cells (Dun and Gao, 2019). Tanshinone IIA and cryptotanshinone have cytotoxic and apoptotic effects against HeLa cell lines (Zaker et al., 2017). Cryptotanshinone inhibits the proliferation by inducing G0/G1 phase arrest and apoptosis of cervical cancer HeLa cells (Ye et al., 2010). Dihydrotanshinone I displays an antiproliferative effect on human cervical cancer cells (Luo et al., 2018).

In endometrial cancer, tanshinone I blocks the proliferation of the human endometrial carcinoma HEC-1-A cells in a dose-dependent manner by causing apoptosis and increasing the ROS level (Li Q. et al., 2018). In summary, tanshinones can significantly suppress the proliferation of gynecological cancer cells *in vitro* and *in vivo* by inducing cell cycle arrest and apoptosis.

**FIGURE 2:**
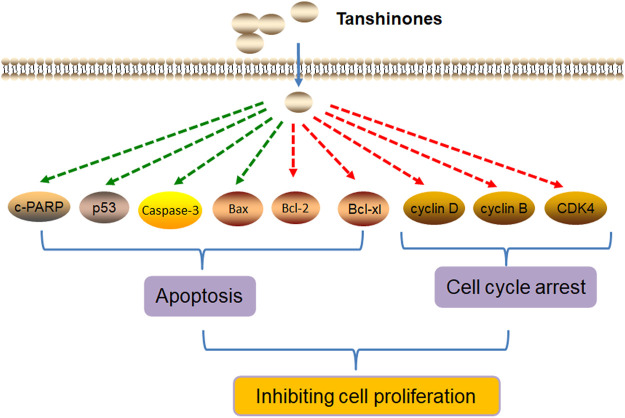
Molecular mechanism of tanshinones inhibiting cell proliferation. Tanshinones inhibit cancer cell proliferation by inducing cell apoptosis and cell cycle arrest. c-PARP, pro-apoptosis–related proteins (cleaved PARP; caspase-3, cysteine aspastic acid–specific protease 3; Bax, Bcl2-associated X protein; Bcl-2, B-cell lymphoma-2; Bcl-xl, Bcl2-associated Xl protein; CDK4, cyclin-dependent kinase 4).

In summary, tanshinones inhibit cell proliferation by inducing cell apoptosis and cell cycle arrest (Figure 2).

## Tanshinones Block Metastasis Phenotype

Invasion and metastasis are one of the main malignant phenotypes of breast cancer cells. It has been found that tanshinone IIA as an active constituent of *Salvia miltiorrhiza* Bunge ameliorates hypoxia-induced epithelial–mesenchymal transition (EMT) in breast cancer MCF-7 cell lines (Fu et al., 2014). Recent studies have shown that tanshinone I significantly downregulates adhesion of MDA-MB-231 cells to human umbilical vein endothelial cells (HUVECs) by reducing ICAM-1 and VCAM-1 expressions in HUVECs and inhibiting the migration of MDA-MB-231 cells through extracellular matrix (Nizamutdinova et al., 2008a). Additionally, tanshinone I inhibits cancer metastasis in the MDA-MB-231 xenografted animal model (Nizamutdinova et al., 2008a). Except nature products, some analogs of tanshinones also exhibit antitumor effects. For example, tanshinone-IIA–based analogs of imidazole alkaloid can effectively inhibit the migration and invasion of MDA-MB-231 cells and metastasis of MDA-MB-231 cells in zebrafish xenografts (Wu et al., 2018). Other studies showed that 2-phenyl-1H-imidazole–based tanshinone IIA derivatives inhibit the invasion and migration of breast cancer cells and metastasis of MDA-MB-231 cells in xenografts. In summary, tanshinone and its derivatives can inhibit invasion, migration, and EMT *in vitro* and metastasis of breast cancer cells *in vivo*.

In ovarian cancer, Zhou et al. found that tanshinone IIA inhibits the migration of A2780 and ID-8 ovarian cancer cells by inhibiting focal adhesion kinase phosphorylation (Zhou et al., 2020c). Another bioactive component, namely, cryptotanshinone, can suppress migration and invasion of ovarian cancer A2780 cells by dramatically inhibiting MMP-2 and MMP-9 expression (Jiang et al., 2017). Wang et al. reported that dihydrotanshinone I can suppress the migration and invasion of ovarian cancer cells in a concentration-dependent manner by downregulating phosphatidylinositol-4,5-bisphosphate 3-kinase catalytic subunit alpha (PIK3CA) (Wang et al., 2020).

In cervical cancer, tanshinone IIA inhibits cervical cancer stem-like cells’ migration and invasion in dose- and time-dependent manners by reducing YAP mRNAs’ stability and transcriptional activity (Qin et al., 2018).

**FIGURE 3:**
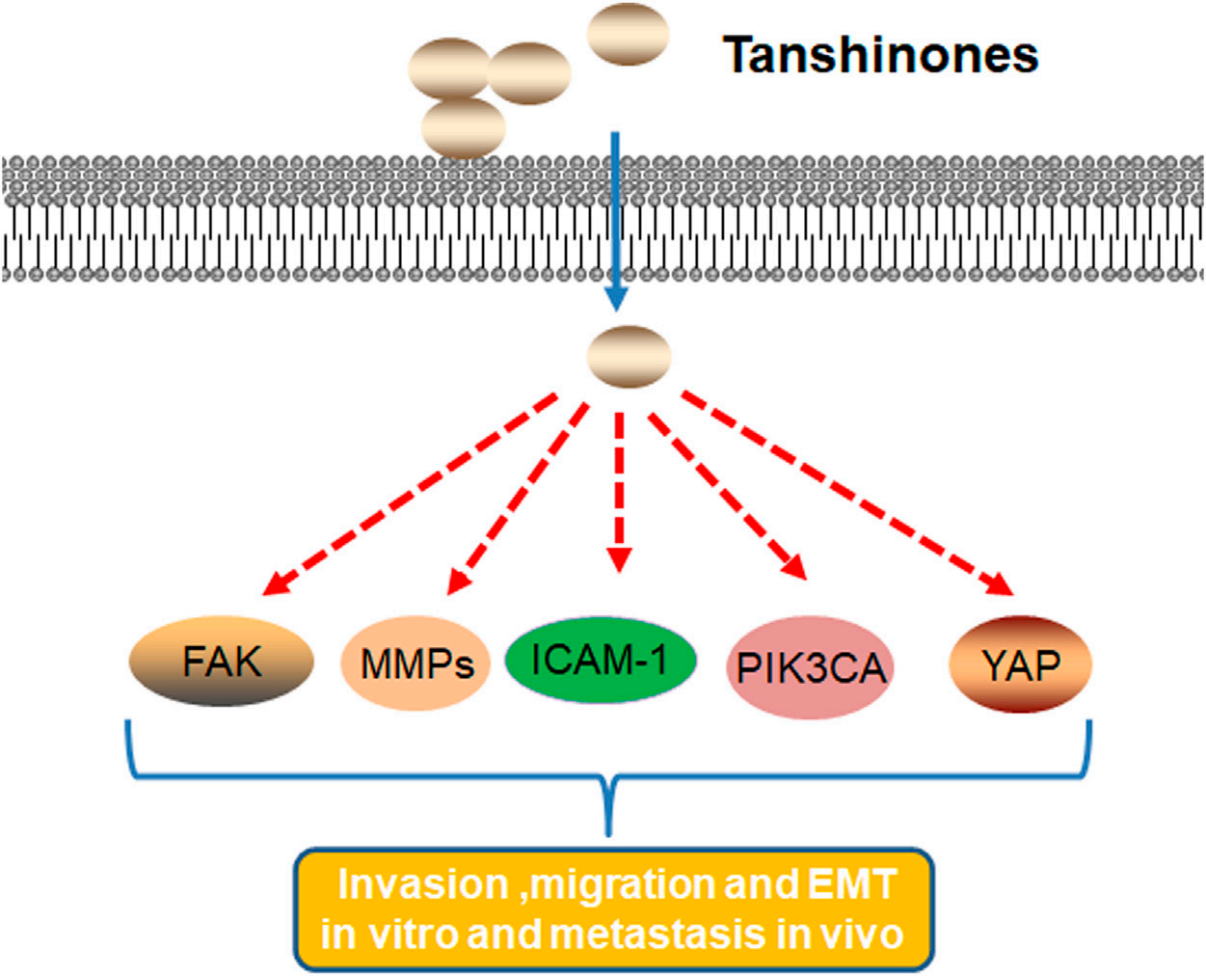
Molecular mechanism of tanshinones inhibiting cell migration, invasion, metastasis, and EMT. Tanshinones inhibit protein expression in migration, invasion, metastasis, and EMT. FAK, focal adhesion kinase; MMPs, matrix metalloproteinases; ICAM-1, intercellular cell adhesion molecule-1; PIK3CA, phosphatidylinositol-4,5-bisphosphate 3-kinase catalytic subunit alpha; YAP, Yes1 associated transcriptional regulator.

In summary, tanshinones block migration, invasion, metastasis, and EMT by downregulating gene expression such as FAK, MMPs, ICAM-1, PIK3CA, YAP, and Yes1 (Figure 3).

## Tanshinones Enhance Chemosensitivity and Radiosensitivity

Chemotherapy is widely employed in clinical treatment of breast cancer, such as cisplatin, paclitaxel, mitoxantrone, gemcitabine antimetabolites, antitumor antibiotics, platinum analogs, and natural anticancer agents. The development of chemoresistance is the main reason for treatment failure. Recent studies have shown that nature agents and Chinese medicine play a critical role in improving the sensitivity of cancer cells to chemotherapeutic drugs. Tanshinone IIA ameliorates hypoxia-induced doxorubicin (Dox) resistance on breast cancer MCF-7 cells (Fu et al., 2014). Tanshinone IIA promotes the chemosensitivity of breast cancer MCF-7 cells to Dox in a dose-dependent manner by downregulating the expression of efflux ABC transporters including P-gp, BCRP, and MRP1 (Li and Lai, 2017). Tanshinone IIA increases chemosensitivity of ER-positive breast cancer MCF-7 cells to chemotherapy agent Taxol by inhibiting Tau expression (Lin et al., 2018). Tanshinone IIA can also promote sensitivity of breast cancer cells to Dox by suppressing the PTEN/AKT pathway and the expression of efflux ABC transporters including P-gp, BCRP, and MRP1 (Li K. et al., 2019). Tanshinone IIA promotes the chemosensitivity of breast cancer MCF-7 cells to Dox by inhibiting β-catenin nuclear translocation (Li et al., 2021). Recent studies have shown that tanshinone IA exhibits MDR reversing potential to human ER-negative breast cancer cells by downregulating BCRP/ABCG2 expression in cancer cells (Jing et al., 2007). Therefore, tanshinone, as an effective chemosensitizer, is used in combination with chemotherapeutic drugs in the treatment of breast cancer.

Tanshinone IIA promotes TRAIL sensitization by upregulating DR5 through the ROS-JNK-CHOP signaling axis in human ovarian carcinoma cell lines (Chang et al., 2015). Our study showed that natural compound tanshinone I enhances the efficacy of paclitaxel chemotherapy on ovarian cancer (Zhou et al., 2020b). Other studies confirmed that cryptotanshinone can sensitize ovarian cancer cells to chemotherapy drug cisplatin (Jiang et al., 2017). In summary, tanshinones facilitate chemotherapy susceptibility in ovarian carcinoma cell to supply the potential clinical use of ovarian carcinoma cells.

Dihydrotanshinone I as the radiosensitizer significantly increases the efficacy of irradiation on HeLa cells by upregulation of p21 expression and caspase-3 activity and downregulation of cyclin B1, p34 (cdc2) expression, which has potential in the treatment of human cervical cancer (Luo et al., 2018).

**FIGURE 4:**
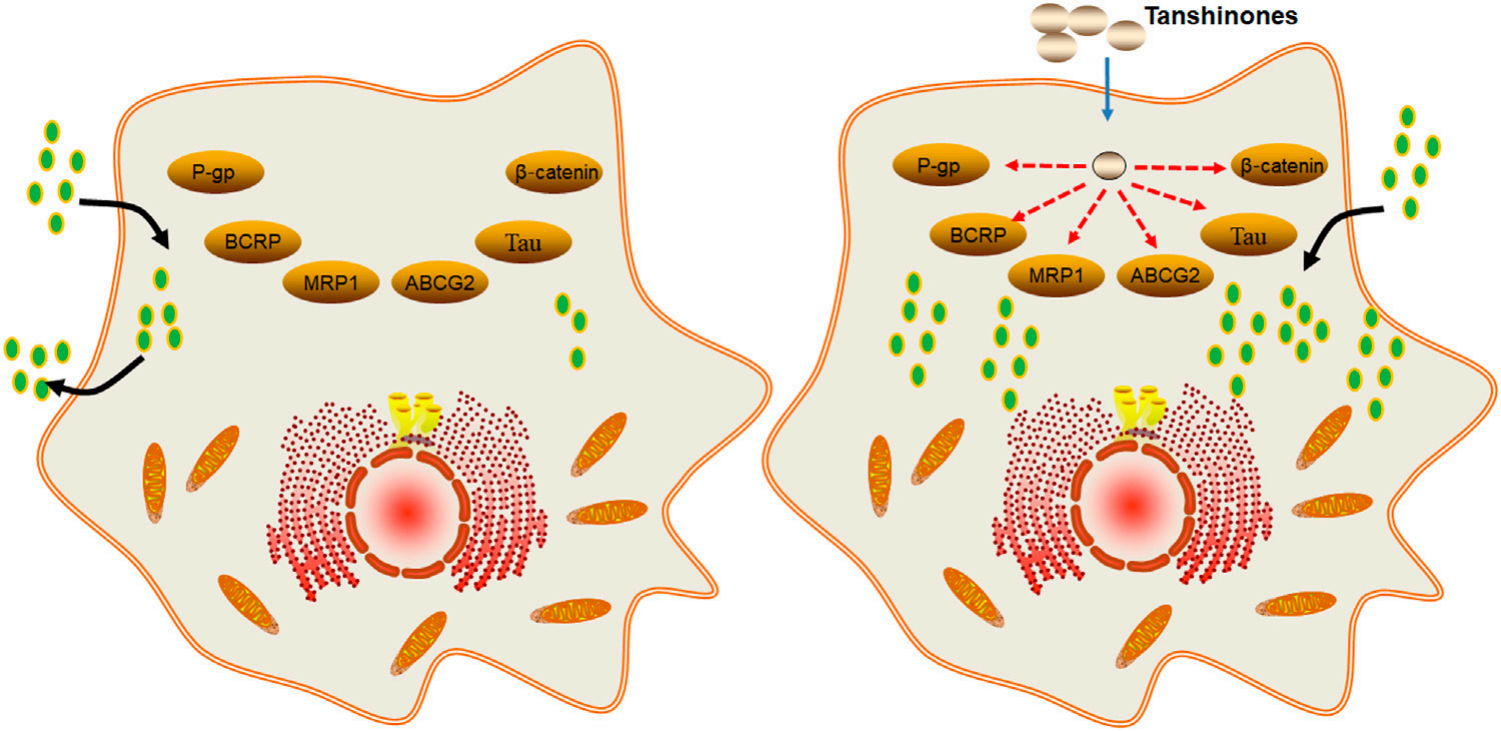
Molecular mechanism of tanshinones increasing the efficacy of chemotherapy and radiotherapy. Tanshinones inhibit protein expression of P-gp, BCRP, MRP1, ABCG2, Tau, and β-catenin. P-gp, P-glycoprotein; multidrug resistance-associated protein 1; ABCG2, ATP binding cassette subfamily G member 2; Tau, microtubule-associated protein; β-catenin, beta-cadherin–associated protein.

In summary, tanshinones can enhance the chemosensitivity and radiosensitivity by reducing the expression of efflux ABC transporters such as P-gp, BCRP, and MRP1 (Figure 4).

## Tanshinones Inhibit Angiogenesis

Angiogenesis plays a key role in tumor proliferation and metastasis and contributes to the cancer progression (Folkman, 2002; Yousefi et al., 2021). To provide enough oxygen and nutrients to tumor growth, tumor vessels are regulated by multiple angiogenic growth factors such as vascular endothelial growth factor (VEGF), basic fibroblast growth factor (bFGF), and matrix metalloproteinases (MMPs) (Jiang et al., 2020; Ma et al., 2020). Recent studies have shown that the inhibition of angiogenesis by drug or antibody-targeting angiogenic growth factors has a potential therapeutic effect on many solid tumors (Pang et al., 2010; Yang M. et al., 2021). Clinical studies have shown that antiangiogenic therapy may have the greatest initial impact on tumor growth and metastasis (Heier et al., 2016; Scott et al., 2017). A number of studies have found that Chinese traditional medicines such as Qingdu granule, Oridonin (from *Rabdosia rubescens*), Ginsenoside Rd (from *Panax ginseng* C. A. Mey.), Arctigenin (from *Arctium lappa* L.), and cyperenoic acid (from *Croton crassifolius* Geiseler) can inhibit the tube formation of HUVECs *in vitro* and exhibit antiangiogenesis of breast cancer *in vivo* by inhibiting the expression of angiogenic growth factors and HIF-1α/VEGF signal pathway (Wang et al., 2017; Zhang et al., 2017; Li C. et al., 2018; Zhao et al., 2018). Tanshinone IIA is one of the major components from Chinese traditional medicine Danshen. Tanshinone IIA inhibits the migration and tube formation of bone marrow–derived endothelial progenitor cells (EPCs) *in vitro* and downregulates VEGF-mediated angiogenesis in the chick embryo chorioallantoic membrane (CAM) model (Lee et al., 2017). Studies showed that tanshinone IIA reduces the expression level of proangiogenic factors such as VEGF and COX2 to inhibit angiogenesis in human colorectal cancer (Zhou et al., 2012; Sui et al., 2017; Zhou L. et al., 2020). In ovarian cancer, tanshinone IIA significantly downregulates VEGF and cyclooxygenase-2 (COX2) mRNA expression *in vitro* and *in vivo* (Zhou et al., 2020c). Tanshinone IIA and tanshinone I suppress angiogenesis by targeting the protein kinase domains of VEGF/VEGFR2 and reducing the expression of VEGF in the human lung cancer cells (Tung et al., 2013; Xie et al., 2015).

In breast cancer, tanshinone I downregulates hypoxia-inducible factor 1alpha (HIF-1α) and VEGF in breast cancer MDA-MB-231 cells, and tanshinone IIA inhibits the angiogenesis and growth of human breast cancer xenografts in nude mice through suppression of HIF-1α and VEGF (Nizamutdinova et al., 2008a; Liao et al., 2020). Cryptotanshinone is one of the tanshinones isolated from the roots of the plant *Salvia miltiorrhiza* Bunge (Danshen). It has been shown that it downregulates the levels of VEGF mRNA and protein in a dose-dependent manner in U2OS osteosarcoma cells and resists angiogenesis ability by inhibiting the tube formation assay *in vitro* (Xu et al., 2016). Therefore, active components from *Salvia miltiorrhiza* Bunge (Danshen) had antiangiogenic effects by inhibiting mRNA and protein expression of proangiogenic growth factors such as VEGF and COX2. Our study showed that tanshinone IIA inhibits angiogenesis by reducing VEGF and COX2 mRNA expression of ovarian cancer cells *in vitro* and *in vivo* (Zhou et al., 2020c).

**FIGURE 5:**
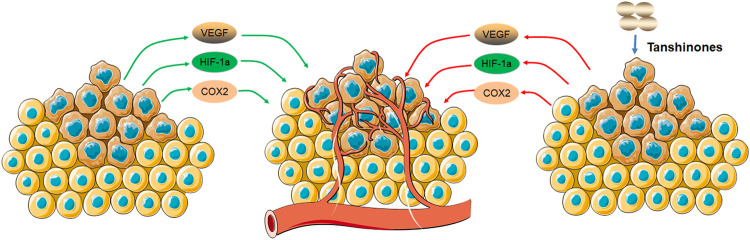
Molecular mechanism of tanshinones inhibiting angiogenesis. Tanshinones inhibit protein expression of VEGF, COX2, and HIF-1α. VEGF, vascular endothelial growth factor; COX2, cytochrome C oxidase II; HIF-1α, hypoxia-inducible factor 1 subunit alpha.

In summary, tanshinones can inhibit cell growth and tumor growth by regulating the expression of proangiogenic growth factors and hypoxia-inducible factor 1alpha (HIF-1α) (Figure 5).

## Conclusion and Expectation

During the past few decades, many studies have demonstrated the potential benefits of tanshinones for the treatment of gynecological tumors *in vitro* and *in vivo*. Mechanism studies showed that tanshinones inhibit cancer cell proliferation and tumor growth by inducing cancer cell apoptosis and cell cycle arrest, inhibiting metastasis phenotype, enhancing chemosensitivity and radiosensitivity, and blocking the angiogenesis of tumor. In addition to the characteristics of malignant proliferation, strong invasion and metastasis, and drug resistance, metabolic reprogramming is another main feature of tumor cells and also the hotspot and focus of tumor research. Metabolic reprogramming of tumor cells refers to the raw materials needed by cells to change their metabolic phenotype in harsh environment, so as to quickly produce adenosine triphosphate (ATP), synthesize macromolecular substances, maintain the redox state of cells, and finally realize the proliferation of tumor cells. Metabolic reprogramming of tumor cells enhances the restriction on glucose and amino acids, obtains nutrients for cell survival through other ways, uses glycolysis and tricarboxylic acid cycle intermediates for biosynthesis, and changes key metabolic enzymes of cell metabolism. Although not all tumor tissues have these characteristics, studies have found that abnormal metabolic phenotypes such as glycolysis, fatty acid metabolism, and glutamine decomposition generally exist in tumor cells. Our study showed that tanshinone I can regulate metabolic reprogramming of tumor cells. However, the specific molecular mechanism is not completely clear. In the future, the antitumor molecular mechanism of tanshinones will be further expanded to provide a new basis for the clinical application of tanshinones.

In addition, cryptotanshinone, tanshinone I, dihydrotanshinone I, and tanshinone IIA have low aqueous solubility and poor membrane permeability, which affected drug absorption (Hao et al., 2006; Wang et al., 2007; Wu et al., 2018). Many studies have demonstrated that tanshinones have poor bioavailability by oral administration or the gavage method (Zhang et al., 2006). For example, the bioavailability of cryptotanshinone in rats was approximately 2.1% by oral administration and 10.6% by intraperitoneal administration (Zhang et al., 2006). Based on these limitations, it is necessary to optimize tanshinone’s structure and increase antitumor potency and drug-like properties. A recent study identified two tanshinone analogs, which increased antitumor potency as compared to the natural tanshinones both Tan-I and Tan-IIA (Gain et al., 2021).

In summary, tanshinones have shown many antitumor mechanisms and have higher potential in the treatment of female breast cancer and gynecological cancer.
